# Autofluorescence imaging reveals the impact of cryopreservation on T cell metabolism and activation response

**DOI:** 10.1016/j.omta.2026.201704

**Published:** 2026-02-19

**Authors:** Dan L. Pham, Meghana Kalluri, Cole Weaver, Angela Hsu, Amani Gillette, Wenxuan Zhao, Tyce Kearl, Peiman Hematti, Nirav N. Shah, Melissa C. Skala

**Affiliations:** 1 Morgridge Institute for Research, Madison, WI 53715, USA; 2 Department of Biomedical Engineering, University of Wisconsin-Madison, Madison, WI 53706, USA; 3 Department of Cellular and Molecular Pathology, University of Wisconsin-Madison, Madison, WI 53726, USA; 4 Blood and Marrow Transplant & Cellular Therapy Program, Division of Hematology & Oncology, Medical College of Wisconsin, Milwaukee, WI 53226, USA

## Abstract

Cryopreservation or the process of freezing cells is a cornerstone of most cell therapy protocols. Optimization of cryopreservation protocols and cryoprotectant agents to improve cell viability and functionality is under further investigation. However, the impact of cryopreservation on cellular metabolism and function immediately post-thaw is not fully understood. Here, we used label-free, non-invasive optical metabolic imaging (OMI) of NAD(P)H and FAD to characterize the activation response of frozen T cells from healthy donors and lymphoma patients post-thaw. Using OMI, we identified significant metabolic shift, along with delayed and diminished activation response, in healthy donor T cells throughout the first 4.5 h upon thawing. In cryopreserved peripheral T cells from lymphoma patients in our bispecific CD20/CD19 CAR T clinical trial, OMI could identify early metabolic stress and allowed gating of metabolically fit cells associated with post-thaw viability. Notably, in our pilot study involving four patients, metabolically fit T cells from complete responders exhibited metabolic responses to activating stimuli within the first 4.5 h post-thaw. Overall, our findings suggest that 4–5 h post-thaw is a critical time window to assess the impact of cryopreservation and thawing, supporting the potential of OMI to optimize cryopreservation protocols and evaluate patient T cell quality for cell therapy.

## INTRODUCTION

Cryopreservation is commonly used for long-term preservation of cells, tissue, and other biological samples, which involves using low temperature (−80°C to −196°C) to slow down cell metabolism and other functional activity. Cryopreservation plays a central role for several cell therapies including chimeric antigen receptor (CAR) or engineered T cell receptor (TCR) therapy. The complex manufacturing process of these cell therapies requires specialized facilities and expertise and often employs a central manufacturing model that relies on cryopreservation of both the starting materials and final products.^1^ For instance, current commercial CAR T manufacturing workflows start with collection of leukapheresis from the patient at the hospital site as the starting material. Patient’s leukapheresis products are transported to the manufacturing facility, where T cells are activated, transduced to express CAR transgene, and expanded.^2^ At the end of the manufacturing process, the CAR T cell product is cryopreserved. This allows for preservation of the product while necessary quality control testing is performed and for transportation to the hospital for infusion into the patient. Despite ongoing research to define the optimal cryopreservation protocol such as cryoprotectant agents, cooling rate, and post-thaw condition, the successful recovery of cell viability and function upon thawing remains challenging.^2,3^ Meanwhile, due to the toxic effect of residual cryoprotectant agent, such as dimethyl sulfide (DMSO), on immune cell viability and effector functions,^4^ most CAR T cell products mandate infusion within 30–90 min after thawing.^5^ Since proper T cell function is critical for the efficacy of CAR T cell therapy, it is important to characterize the impact of cryopreservation on T cells within a short time frame post-thaw, particularly concerning T cell activation response, a key function for CAR T manufacturing success and clinical outcome.

During CAR T cell manufacturing, T cell activation is induced by targeting stimulatory and co-stimulatory receptors on the T cell surface, such as CD3, CD28, and CD2.^6^ Activation primes T cells for *CAR* gene transfer while followed by a proliferation phase to achieve a sufficient CAR T cell dosage, both of which are required for successful CAR T cell manufacturing.^7^ Most CAR T products are frozen while going under quality control testing before their release. Antigen-specific activation of CAR T cells upon thawing and infusion into the patient and interaction with target tumor cells initiates cytotoxic functions to eliminate cancer cells, leading to tumor regression and patient treatment response. Therefore, understanding how cryopreservation affects CD3-mediated and antigen-specific activation of T cells upon thawing will allow optimization of cryopreservation protocols that ultimately enhance CAR T cell performance.

For T cell activation, metabolic reprogramming is crucial, not only to provide the necessary energy and molecules for effector function but also to shape the epigenetic landscape that determines T cell differentiation fate.^8–10^ Cryopreservation has been shown to affect cell viability and metabolism-related proteins while reducing metabolic activity in various mammalian single-cell or multi-cellular models such as embryos and other reproductive cells,^11–13^ engineered tissues (osteoblasts^14^), and transplanted hepatocytes^15,16^ or pancreatic islets.^17,18^ However, the impact of cryopreservation is cell-type-dependent,^19^ and immediate metabolic changes in T cells post-thaw and their implication on early activation response remain unclear. We propose that continuous monitoring of T cell metabolism in real time throughout a short window immediately post-thaw and in response to activation stimuli could provide essential insight for future improvements in the freeze-thaw processes.

While several bioassays exist for metabolism and T cell activation assessments, most of them either require destructive and time-consuming sample preparation that is not suitable for frequent analysis of the same culture over time or do not offer single-cell resolution to characterize heterogeneous cell function. To address these challenges, we have developed optical metabolic imaging (OMI), a label-free non-invasive imaging method adapted from fluorescence lifetime imaging microscopy (FLIM) techniques that allows assessment of single-cell metabolism based on autofluorescent signals of metabolic coenzymes NAD(P)H and FAD.^20–22^ NAD(P)H and FAD are an electron donor and acceptor, respectively, and are essential components of many metabolic pathways, including those important for T cell function and activation response such as glycolysis.^23,24^ OMI yields 13 cellular metabolic features based on fluorescence intensity and lifetime of NAD(P)H and FAD, including NAD(P)H intensity, τ_m_, τ_1_, τ_2_, α_1_, and α_2_; FAD intensity, τ_m_, τ_1_, τ_2_, α_1_, and α_2_; the optical redox ratio (ORR); and cell size. ORR is defined as the ratio of NAD(P)H intensity over the sum of NAD(P)H intensity and FAD intensity, which has been correlated to the cellular redox balance.^25,26^ Meanwhile, fluorescence lifetime is defined as time taken for a molecule in the excited state to decay back to the ground state and emit a fluorescence signal. Free NAD(P)H self-quenches, resulting in a short fluorescence lifetime of around 400 ps.^27,28^ Upon protein-binding, NAD(P)H undergoes conformational changes, leading to long fluorescence lifetime of around 2.2–2.5 ns.^28,29^ FAD displays a reversed trend in fluorescence lifetime, with free FAD having a long lifetime and bound FAD having a short lifetime.^24,26^ Therefore, the fluorescence lifetimes of NAD(P)H and FAD are indicative of their binding activity.

Previously, we demonstrated that metabolic imaging based on autofluorescence from NAD(P)H and FAD is sensitive and specific in characterizing metabolic changes upon T cell activation.^30,31^ Here, we used OMI to determine the impact of cryopreservation on metabolism and activation response in T cells from healthy donors and patients with B cell malignancies, along with implications of cryopreservation for adoptive T cell therapy. Using OMI, we also evaluated the metabolic response accompanying different modes of T cell activation, including CD3-driven and antigen-specific activation in cryopreserved cells.

## RESULTS

### OMI identifies significant changes in frozen T cell metabolism from healthy donors upon thawing

**Figure 1.**
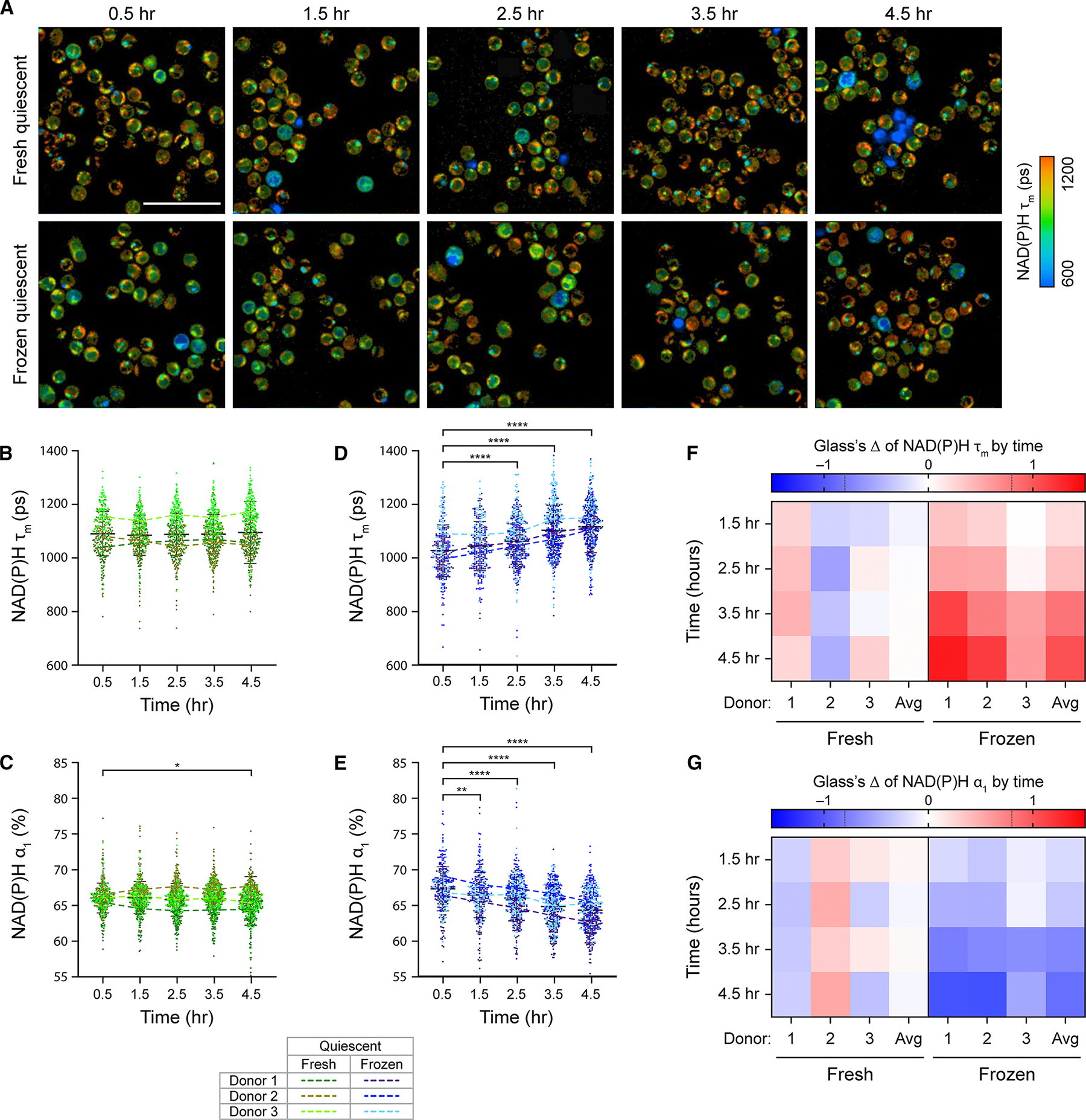
Cryopreserved T cells underwent significant metabolic changes upon thawing (A) Representative NAD(P)H mean lifetime (NAD(P)H τ_m_) images of donor-matched fresh (top) and frozen (bottom) quiescent CD3 T cells throughout a 4.5-h imaging time course. (B–E) Quantification of NAD(P)H τ_m_ and free NAD(P)H proportion (NAD(P)H α_1_) of fresh (B and C) or frozen (D and E) quiescent T cells. *n* = 299–537 cells/condition/time point across three biologically independent donors. Dots represent individual cells, color coded by condition (fresh or frozen) and donor. Two-sided non-parametric Kruskal-Wallis test with Dunn’s post hoc test for multiple comparisons against corresponding OMI measurements at the 0.5-h time point. (F and G) Glass’s Δs to quantify effect sizes of changes over time in (F) NAD(P)H τ_m_ and (G) NAD(P)H α_1_ of fresh and frozen quiescent T cells, with respect to the corresponding OMI measurements at the 0.5-h time point. Glass’s Δ was reported for individual donors and the average values across three donors (Avg). Left 4 columns: fresh quiescent T cells, right 4 columns: frozen quiescent T cells. Black dashed lines on Glass’s Δ color bar represent |Glass’s Δ| = 0.8, threshold for strong effect size. Scale bars, 50 μm. Bars are mean ± standard deviation. *p < 0.05, **p < 0.01, ****p < 0.0001.

CD3 T cells were isolated from peripheral blood of three healthy donors and divided in half for cryopreservation and fresh culture for 24 h. Upon thawing, cryopreserved and fresh T cells from matched donor were imaged simultaneously using OMI every hour (Figure 1A). Throughout the 4-h imaging time course, fresh quiescent T cells displayed stable metabolism, with no significant changes in NAD(P)H mean lifetime (NAD(P)H τ_m_) and a slight increase in the proportion of free NAD(P)H (NAD(P)H α_1_) at the 4.5-h time point (Figures 1B and 1C). Interestingly, we observed significant and consistent metabolic changes in frozen quiescent T cells upon thawing (Figures 1D and 1E). Across three donors, frozen T cells showed a gradual but significant increase in NAD(P)H τ_m_ and decrease in NAD(P)H α_1_ within the first 4.5 h post-thaw. To determine the effect sizes of metabolic changes over time, we calculated Glass’s Δ at each time point with respect to the 0.5-h time point (Figures 1F and 1G). Glass’s Δ revealed a small effect of imaging time on fresh quiescent T cell metabolism. For several OMI parameters, the average effect size across three donors at each time point remain within −0.1 to 0.1, further indicating stable metabolism in fresh quiescent T cells (Figures 1F and 1G, left). Meanwhile, we observed a large effect of time post-thaw on frozen quiescent T cell metabolism. Over time, Glass’s Δ for several OMI parameters of frozen T cells increased and reached an average value of greater than 1 (for NAD(P)H τ_m_) or smaller than −1 (for NAD(P)H α_1_) at the 4.5-h time point (Figures 1F and 1G, right). This indicated that, for frozen quiescent T cells, the differences in these OMI parameters exceeded one standard deviation throughout the imaging time course. Using OMI, we have determined consistent and significant metabolic changes in frozen T cells, with a shift toward increasing NAD(P)H τ_m_ and decreasing NAD(P)H α_1_ that continued up to 4.5-h post thaw.

### Fresh and frozen T cells from healthy donors displayed distinct metabolic response to activation during the first 4.5 h

**Figure 2.**
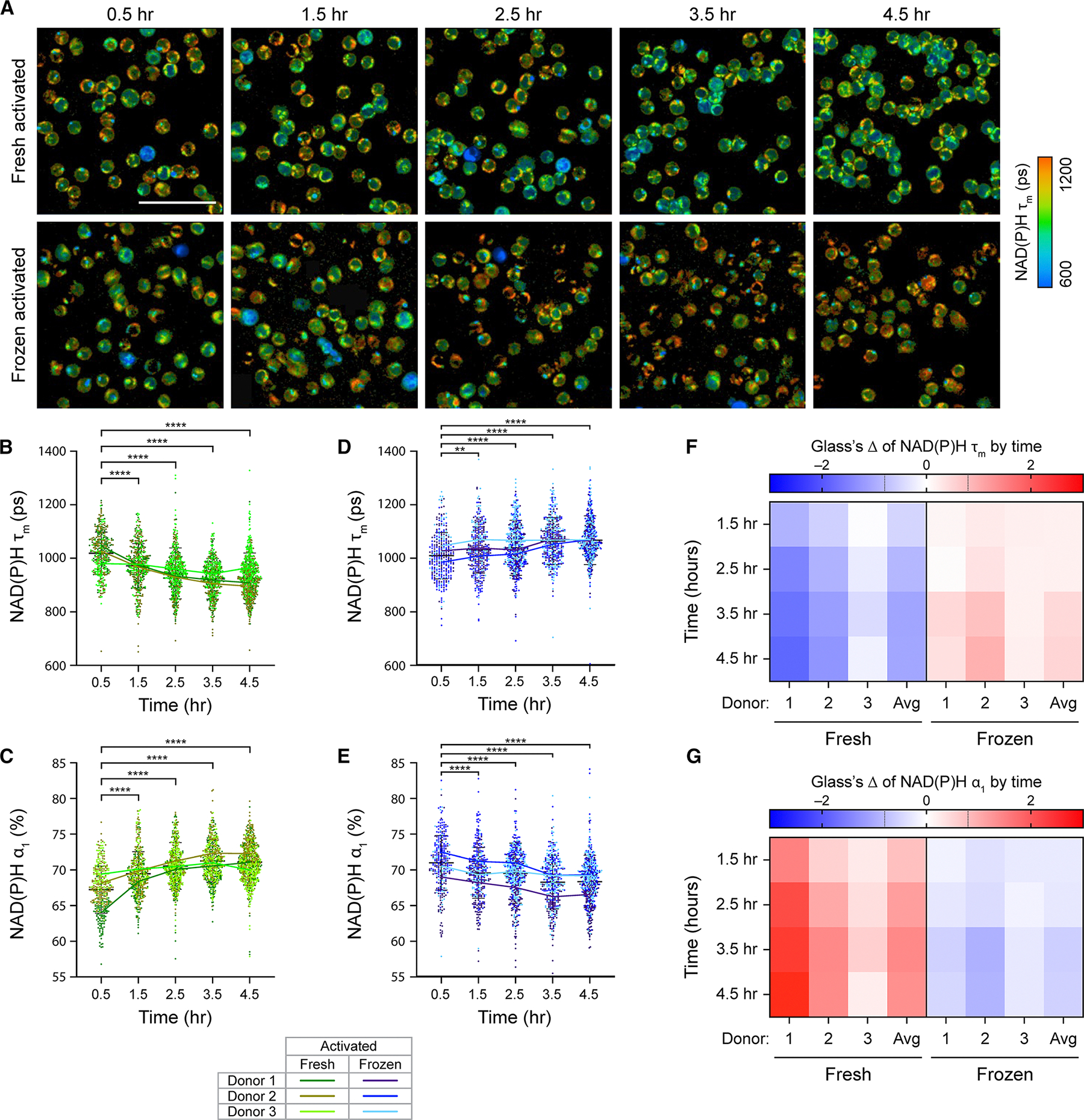
Fresh and frozen T cells displayed distinct metabolic response toward activating stimulus (A) Representative NAD(P)H τ_m_ images of donor-matched fresh (top) and frozen (bottom) activated T cells upon activation. Frozen T cells were activated immediately after thawing. (B–E) Quantification of NAD(P)H τ_m_ and NAD(P)H α_1_ of (B and C) fresh or (D and E) frozen activated T cells. *n* = 296–618 cells/condition/time point across three biologically independent donors. Two-sided non-parametric Kruskal-Wallis test with Dunn’s post hoc test for multiple comparisons against corresponding OMI measurements at the 0.5-h time point. (F and G) Glass’s Δs to quantify effect sizes of changes over time in (F) NAD(P)H τ_m_ and (G) NAD(P)H α_1_ of fresh and frozen activated T cells, with respect to the corresponding OMI measurements at the 0.5-h time point. Glass’s Δ was reported for individual donors and the average values across three donors (Avg). Black dashed lines on Glass’s Δ color bar represent |Glass’s Δ| = 0.8, threshold for strong effect size. Left 4 columns: fresh activated T cells, right 4 columns: frozen activated T cells. Scale bar is 50 μm. Bars are mean ± standard deviation. *p < 0.05, **p < 0.01, ****p < 0.0001.

Due to significant changes in frozen T cell metabolism upon thawing, we further investigated how cryopreservation impacts T cell activation response using OMI. Frozen T cells from three healthy donors were immediately activated with StemCell αCD2/αCD3/αCD28 upon thawing (time = 0 h). Similarly, donor-matched fresh T cells were also activated at the same time (0 h) (Figure 2A). In fresh T cells, we observed the characteristic metabolic changes, with low NAD(P)H τ_m_ and high NAD(P)H α_1_, following activation (Figures 2B and 2C). This is consistent with previous studies showing a shift toward glycolysis in activated T cells.^8,9^ These changes in T cell metabolism occurred early and were significant within 1 h of activation. Activation-induced metabolic changes in fresh T cells continued to progress, with decreasing NAD(P)H τ_m_ and increasing NAD(P)H α_1_ observed over time (Figures 2B and 2C). We did not, however, observe similar metabolic changes in frozen activated T cells (Figures 2D and 2E). Across three donors, frozen activated T cells showed increased NAD(P)H τ_m_ and decreased NAD(P)H α_1_ that were sustained throughout the 4-h imaging time course. These metabolic changes were opposite of donor-matched fresh activated T cells (Figures 2B and 2C) but were consistent with frozen quiescent T cells (Figure 1).

Glass’s Δ with respect to the 0.5-h time point further confirmed opposite metabolic responses of donor-matched activated fresh and frozen T cells over time (Figures 2F and 2G). Throughout activation (0.5–4.5 h), fresh T cells showed large effects in NAD(P)H τ_m_ and NAD(P)H α_1_ that strengthened over time, with average absolute Glass’s Δ values exceeding 1.2 at the 4.5-h time point (Figures 2F and 2G, left). These findings demonstrated that fresh T cell metabolism, especially NAD(P)H binding activity, were highly responsive to activating stimulus. Meanwhile, for frozen T cells, we observed weak- to moderate-effect sizes throughout the imaging time course (Figures 2F and 2G, right). Interestingly, at 4.5 h, average Glass’s Δ with respect to 0.5 h of frozen activated T cells (0.60 for NAD(P)H τ_m_ and −0.68 for NAD(P)H α_1_) were smaller than those of frozen quiescent cells (1.03 for NAD(P)H τ_m_ and −1.01 for NAD(P)H α_1_, respectively). We hypothesized that since thawing (increased NAD(P)H τ_m_ and decreased NAD(P)H α_1_) and activation (decreased NAD(P)H τ_m_ and increased NAD(P)H α_1_) induced opposite changes in NAD(P)H lifetime parameters, the effects of imaging time (or time post thawing and activation) on frozen activated T cells were confounded. However, since we observed an overall similar metabolic change (increased NAD(P)H τ_m_ and decreased NAD(P)H α_1_) in both frozen quiescent and frozen activated T cells within the first 4.5-h post-thaw, our findings suggested that the effect of cryopreservation/thawing was dominant during this time window, and this potentially affected the ability of frozen T cells to respond to activating stimulus.

### OMI revealed delayed activation response in frozen T cells post-thaw

**Figure 3.**
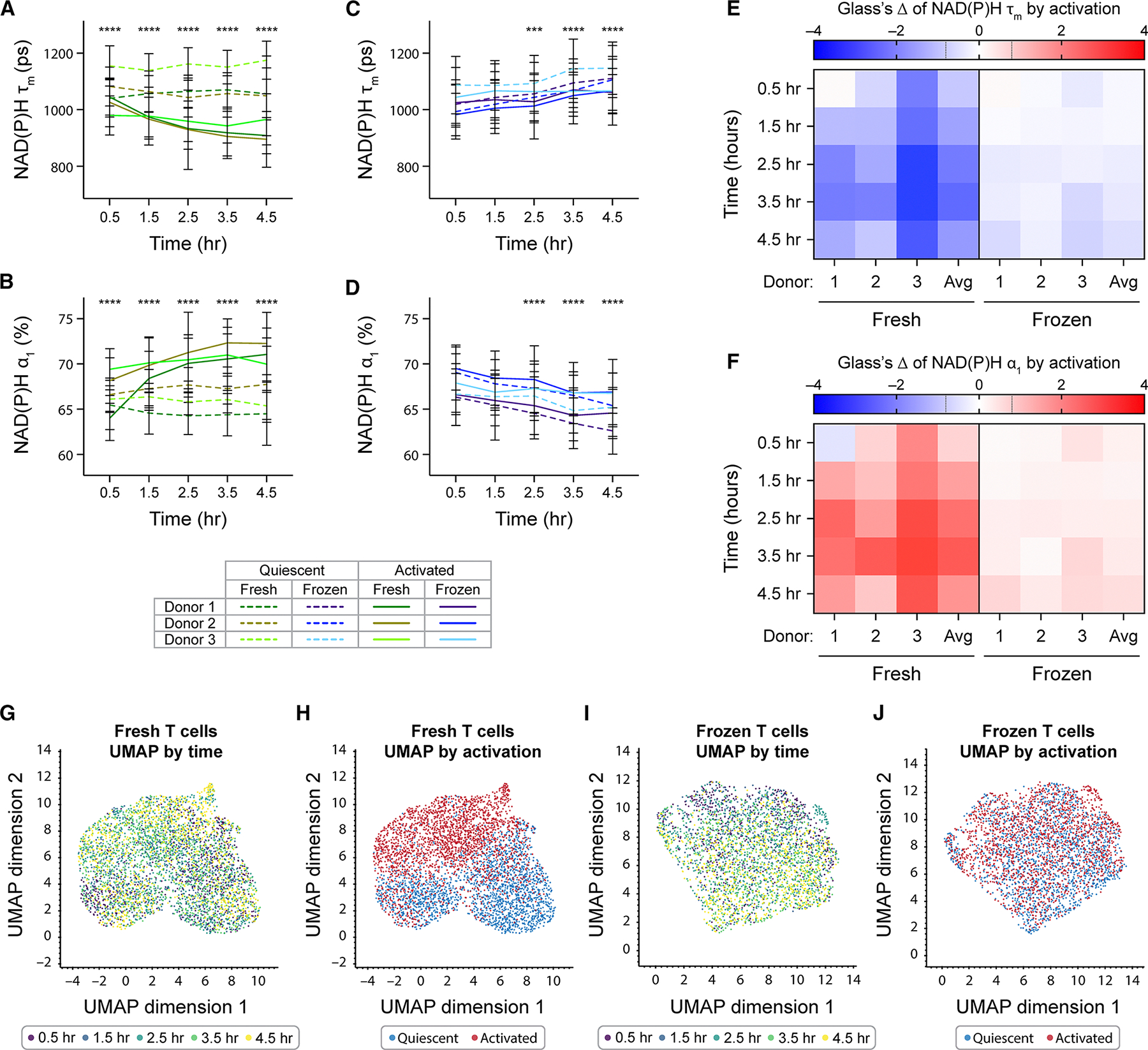
Cryopreservation delayed and diminished activation response in frozen T cells upon thawing (A–D) Quantification of NAD(P)H τ_m_ and NAD(P)H α_1_ of (A and B) fresh or (C and D) frozen T cells from three independent donors. Lines represent donor averages, color coded by condition (fresh or frozen) and donor, while line patterns indicate activation status (dashed line: quiescent, solid line: activated). *n* = 296–618 cells/condition/time point across three donors. ANOVA with three factors: donor (donor 1, 2, and 3), time (0.5–4.5 h), and activation status (quiescent, activated). Tukey post hoc test was used to determine statistical significance for multiple comparisons between quiescent and activated groups at each time point. (E and F) Glass’s Δs to quantify effect size of activation on (E) NAD(P)H τ_m_ and (F) NAD(P)H α_1_ of fresh and frozen T cells over time, with respect to corresponding quiescent group at each time point. (G and H) Uniform manifold approximation and projection (UMAP) of 11 OMI parameters (NAD(P)H and FAD τ_m_, τ_1_, τ_2_, α_1_, α_2_, and cell size) of fresh T cells from three donors, color coded by (G) time and (H) activation status. *n* = 4,595 cells. (I and J) UMAP of 11 OMI parameters of frozen T cells from three donors, color coded by (I) time and (J) activation status. *n* = 3,451 cells. Bars are mean ± standard deviation. Black dashed lines on Glass’s Δ color bar represent |Glass’s Δ| = 0.8, threshold for strong effect size. ***p < 0.001, ****p < 0.0001.

Since we observed different metabolic changes in fresh and frozen T cells upon activation, we further characterized the activation response in these groups compared to their donor-matched quiescent groups. We observed significantly lower NAD(P)H τ_m_ and higher NAD(P)H α_1_ between quiescent and activated fresh T cells as early as 0.5 h after activation. The differences in these NAD(P)H lifetime parameters between fresh quiescent and fresh activated T cells further widened as the activation duration increased (Figures 3A and 3B). We also observed significantly higher normalized NAD(P)H intensity in fresh activated T cells compared to donor-matched fresh quiescent cells (Figure S2A). The increase inNAD(P)H intensity, decrease in NAD(P)H τ_m_, and increase in NAD(P)H α_1_ indicated an increase in free NAD(P)H abundance following activation, as free NAD(P)H is associated with shorter fluorescence lifetime compared to protein-bound NAD(P)H, consistent with previous studies.^32,33^

Compared to fresh T cells, donor-matched frozen T cells displayed smaller and delayed changes due to activation, with no significant differences in NAD(P)H τ_m_ or NAD(P)H α_1_ observed between frozen quiescent and frozen activated T cells until 2.5 h post-activation (Figures 3C and 3D). At the 2.5-h time point, frozen activated T cells displayed significantly lower NAD(P)H τ_m_ and higher NAD(P)H α_1_ compared to frozen quiescent T cells. These differences continued up to 4.5-h post-activation (Figures 3C and 3D). Normalized NAD(P)H intensity of frozen T cells was less responsive to activating stimulus (Figure S2B).

To determine the effect size of activation on fresh and frozen T cells, we calculated the Glass’s Δ at each time point with respect to donor-matched quiescent groups (Figures 3E and 3F). While we observed metabolic changes in a similar direction with activation in both fresh and frozen T cells, specifically lower NAD(P)H τ_m_ and higher NAD(P)H α_1_, the activation effects on fresh T cells (Figures 3E and 3F, left) were consistently greater than on donor-matched frozen T cells (Figures 3E and 3F, right). Activation induced strong effects on NAD(P)H τ_m_ and NAD(P)H α_1_ of fresh T cells, with effect sizes of up to −2.8 and 3.0 within 4.5 h following activation. This indicated a difference of up to three standard deviations between donor-matched activated and quiescent cells. Meanwhile, for frozen T cells, activation effects on NAD(P)H τ_m_ and NAD(P)H α_1_ were smaller and remained within −0.7 to 0.7. Similarly, we observed greater effect size of activation on normalized NAD(P)H intensity of fresh T cells compared to frozen T cells (Figure S2C). However, effect sizes due to activation did increase over time for frozen T cells, with the greatest Glass’s Δ observed at 4.5-h post-activation (Figures 3E and 3F, right). This suggests that as activation duration and time post-thaw increased, the ability of frozen T cells to respond to activating stimulus started to recover.

To understand the metabolic landscape of fresh and frozen T cells during activation, we projected all OMI lifetime parameters of fresh and frozen T cells with different activation statuses on a two-dimensional uniform manifold approximation projection (UMAP) (Figures 3G–3J). We observed the OMI landscape of fresh T cells mainly clustered based on activation rather than by time (Figures 3G, 3H, S2D, and S2E). Meanwhile, frozen T cells demonstrated a shift in clustering pattern from the top left to bottom right corner of the UMAP over the imaging time course, indicating a gradual shift in their metabolism (Figure 3I) but did not display distinct clusters based on activation status (Figures 3J, S2F, and S2G). This suggests that within 4.5 h upon stimulation, OMI captured more robust and significant metabolic changes in response to activation in fresh T cells compared to donor-matched frozen T cells. The distinct OMI metabolic profiles of fresh activated and quiescent T cells also allowed classification by activation status with high sensitivity and specificity (area under the curve [AUC] >0.91) across several classifier models (Figure S2H), which could not be achieved in frozen T cells (AUC <0.68) (Figure S2I). Additionally, we also observed significantly higher production of pro-inflammatory cytokine tumor necrosis factor alpha (TNF-α) by fresh activated T cells compared to donor-matched frozen activated T cells after 4.5 h of stimulation (Figure S2J). Overall, our data suggest a delayed and diminished activation response by frozen T cells compared to fresh T cells upon thawing.

### OMI identified activation in frozen T cells after 48 h of stimulation

**Figure 4.**
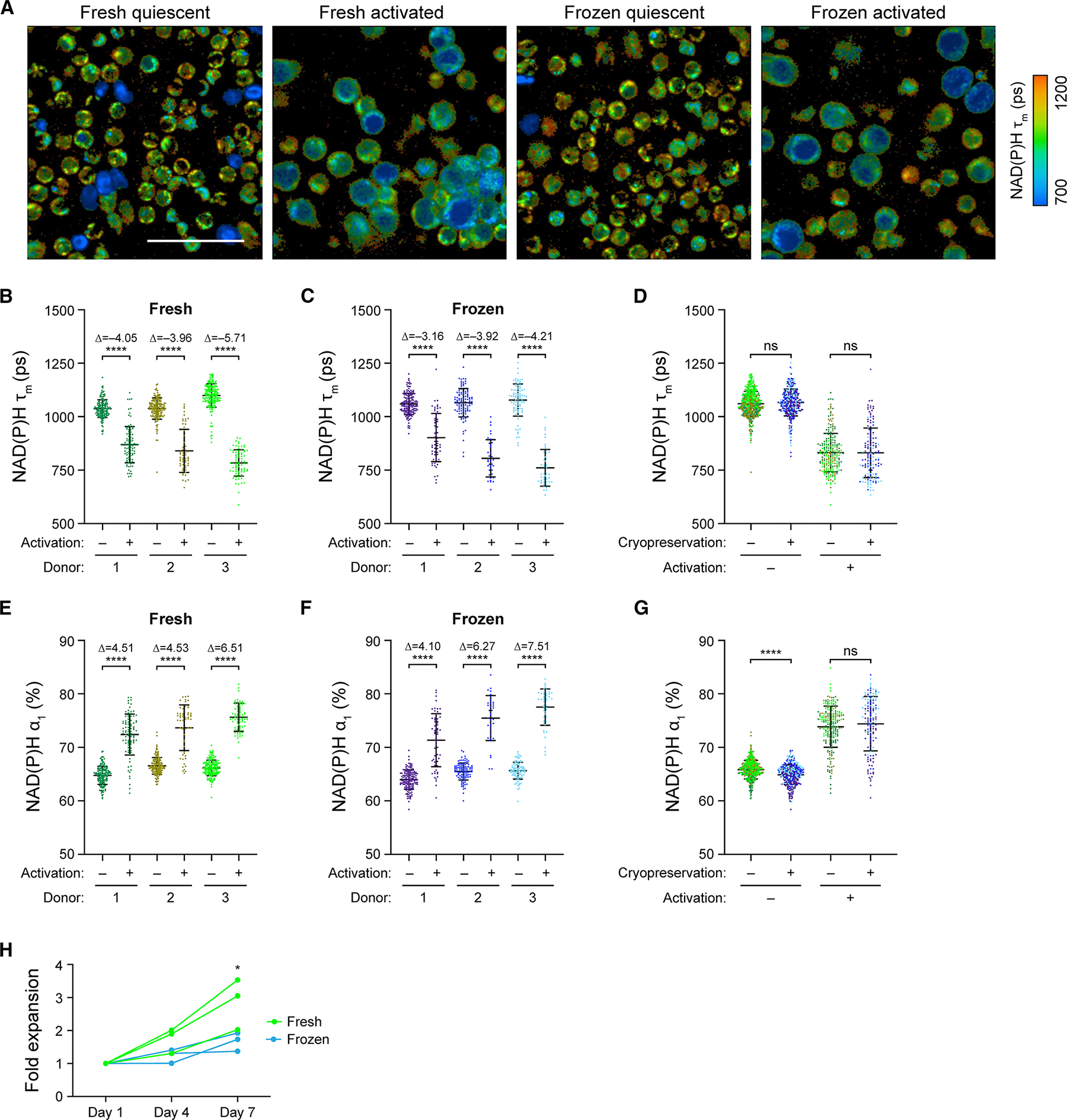
Activation response of frozen T cells recovered after 48 h of activation post-thaw (A) Representative NAD(P)H τ_m_ images of fresh and frozen quiescent and activated T cells at 48 h after activation. (B and C) Quantification and (D) comparison of NAD(P)H τ_m_ of (B) fresh and (C) frozen quiescent and activated T cells at the 48-h time point from three donors. (E and F) Quantification and (G) comparison of NAD(P)H α_1_ of (E) fresh and (F) frozen quiescent and activated T cells at the 48-h time point. For (B–G) *n* = 36–195 cells/condition/donor. Two-sided non-parametric Kruskal-Wallis test with Dunn’s post hoc tests for multiple comparisons between (B, C, E, and F) quiescent versus activated groups for each donor and (D and G) fresh versus frozen for all three donors. (H) Fold expansion through a 7-day expansion time course of fresh and frozen activated T cells from three donors. Two-way repeated measures ANOVA with two factors: day (day 1, 4, and 7) and cryopreservation status (fresh, frozen). Sidak post hoc test for multiple comparisons between fold expansions of fresh versus frozen T cells at each time point. Three data points from three independent donors were counted as repeated measurements for statistical test. Scale bars, 50 μm. Bars are mean ± standard deviation. *p < 0.05, ****p < 0.0001.

As frozen T cells showed delayed and diminished response to activating stimulus within the first 4.5 h post-thaw, we further investigated the impact of cryopreservation and thawing on T cell activation response at a later time point. OMI at 48 h post-activation revealed significant metabolic and morphological differences between quiescent and activated T cells, for both fresh and frozen groups (Figure 4A). We observed significantly lower NAD(P)H τ_m_ (Figures 4B and 4C), higher NAD(P)H α_1_ (Figures 4E and 4F), greater cell size (Figures S3A and S3B), as well as higher ORR (Figures S3D and S3E) in fresh activated and frozen activated T cells compared to donor-matched quiescent cells. There was no significant difference in NAD(P)H τ_m_ between fresh and frozen cells at 48 h; however, frozen activated T cells displayed slightly lower NAD(P)H α_1_ (Figures 4D and 4G). Meanwhile, frozen quiescent T cells demonstrated greater cell size and lower ORR compared to fresh quiescent T cells (Figures S3C and S3F). Overall, donor-matched Glass’s Δ revealed consistently great activation effects (|Glass’s Δ| >3) at 48 h in both fresh and frozen groups (Figures 4B, 4C, 4E, and 4F). These findings suggested that at 48 h after thawing and activation, frozen T cells recovered their activation response. However, we did observe consistently lower fold expansion in frozen T cells compared to their donor-matched fresh counter-parts throughout a 7-day expansion period (Figure 4H), which indicated a potential impact of cryopreservation on T cell expansion capacity.

Binding of stimulatory and co-stimulatory receptors on T cells, such as via CD3/CD28 antibody, provides activation signals for T cells. Meanwhile, antigen-specific activation serves as another mode of activation that is relevant for *in vivo* functions of adoptive cell therapies including CAR and TCR therapies. Therefore, we also further investigated antigen-specific activation response in frozen T cells since cryopreservation is widely used in the context of these adoptive cell therapies. We performed OMI of cytomegalovirus (CMV)-specific T cells activated with StemCell αCD2/αCD3/αCD28 or HLA-matched CMV peptides. We observed similar metabolic response in frozen CMV-specific T cells upon thawing, characterized by increase in NAD(P)H τ_m_ that was sustained up to 5 h post-thaw (Figures S4A–S4D). This is consistent with our data on frozen CD3 T cells reported above. Similarly, over the first 5 h of activation post-thaw, CMV-specific T cells activated with αCD2/αCD3/αCD28 antibody and CMV peptide both showed significant increase in NAD(P)H τ_m_ (Figures S4E and S4F). This suggests a potential impact of cryopreservation and thawing not only on antibody-activation but also on antigen-specific activation response in T cells. Notably, while low NAD(P)H τ_m_ is characteristic of T cell activation response, CMV-specific T cells activated with CMV peptide displayed slightly higher NAD(P)H τ_m_ compared to the quiescent group during the first hour post-thaw (Figures S4G). Interestingly, compared to αCD2/αCD3/αCD28 antibody activation, antigen-specific activation with HLA-matched CMV peptide induced faster and stronger metabolic response in frozen CMV-specific T cells (Figure S4G, pink line versus purple line; Figure S4H, right versus left). Starting at 3 h post-thaw, frozen-CMV-specific T cells activated with HLA-matched CMV peptide (Figure S4G, pink line) showed significantly lower NAD(P)H τ_m_ compared to control quiescent group (Figure S4G, blue line). This difference was sustained up to the 5-h time point, and Glass’s Δs representing antigen-specific activation effects on NAD(P)H τ_m_ strengthened overtime (Δ = −0.03 at 3 h post-thaw, Δ = −0.44 at 5 h post-thaw). Meanwhile, frozen-CMV-specific T cells activated with αCD2/αCD3/αCD28 antibody did not show significantly lower NAD(P)H τ_m_ compared to control group until the 5-h time point (Figure S4G, purple line versus blue line; Figure S4H, left). As T cells produce several inflammatory cytokines such as interferon gamma (IFN-γ) upon activation, we further quantified the amount of IFN-γ secreted by different CMV-specific T cells groups during the 5-h activation time course. Interestingly, antigen-specific activation with CMV peptides resulted in significantly higher IFN- γ production compared to αCD2/αCD3/αCD28 antibody-activated cells and quiescent cells (Figure S4I). This is consistent with the greater changes in NAD(P)H τ_m_ observed in the antigen-activated group. In summary, our data suggest that cryopreservation affects both CD3 receptor activation and antigen-specific activation in T cells. However, antigen-specific activation induces stronger and faster metabolic as well as functional response in post-thaw T cells.

### OMI characterized early metabolic stress in cryopreserved cancer patient T cells post-thaw

**Figure 5.**
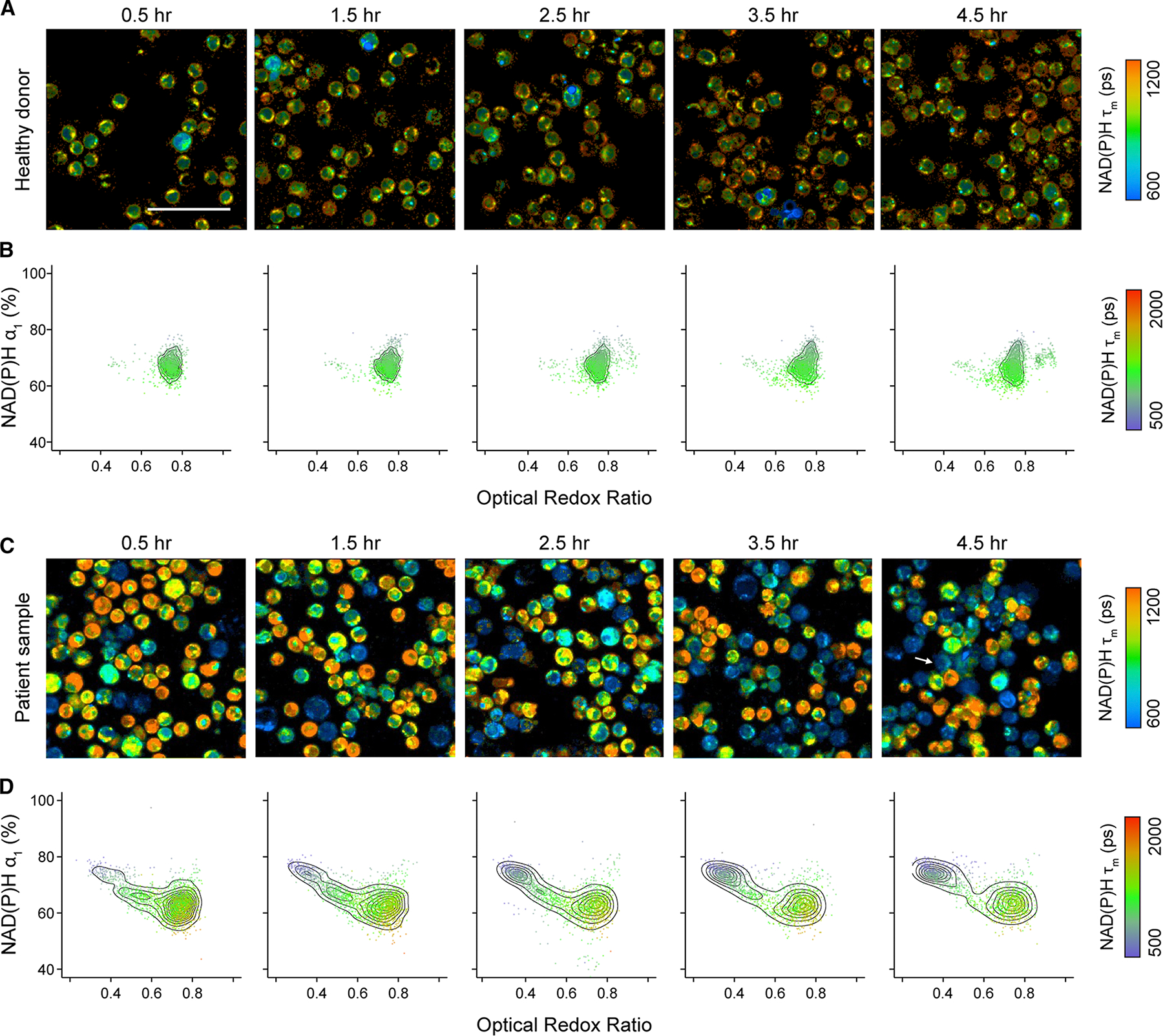
Cryopreserved CD3 T cells from healthy donors and cancer patients exhibited distinct metabolic responses upon thawing (A) Representative NAD(P)H τ_m_ images and (B) two-dimension density contour of NAD(P)H α_1_ and ORR, color coded by NAD(P)H τ_m_ of CD3 T cells from three healthy donors throughout 4.5 h upon thawing. (C) Representative NAD(P)H τ_m_ images and (D) two-dimension density contour of NAD(P)H α_1_ and ORR, color coded by NAD(P)H τ_m_ of CD3 T cells from four patients with either diffuse large B cell lymphoma or mantle cell lymphoma throughout 4.5 h upon thawing. *n* = 8,046 cells for (B) and 1,1921 cells for (D). Scale bars, 50 μm.

Cryopreservation is essential for the storage and distribution of cell therapies. Using OMI, we have shown that short-term cryopreservation (24 h) significantly alters the metabolic state of T cells from healthy donors during the early post-thaw period. Therefore, we aimed to further assess the impacts of cryopreservation on peripheral T cells from four patients with either diffuse large B cell lymphoma (DLBCL) or mantle cell lymphoma (MCL), who would later undergo bispecific CD20/CD19 CAR T cell therapy (Table S1).^34^ A subset of CD3 T cells isolated from these patients as the starting materials for their CAR T therapy were cryopreserved in Cryostor_media containing 5% DMSO at −80°C. Using OMI, we monitored metabolic profiles of these patient T cells immediately after thawing and over the course of 4.5 h post-thaw. We identified distinct metabolic responses in cryopreserved T cells from healthy donors (Figures 5A and 5B) versus lymphoma patients (Figures 5C and 5D). Notably, a second population emerged over time in patient T cells—characterized by a low ORR, increased NAD(P)H α_1_, and reduced NAD(P)H τ_m_—which was not observed in healthy donor T cells (Figures 5B and 5D). This population also exhibited morphological signs of stress, including compromised nuclear and cellular membrane integrity (Figure 5C, white arrow).

**Figure 6.**
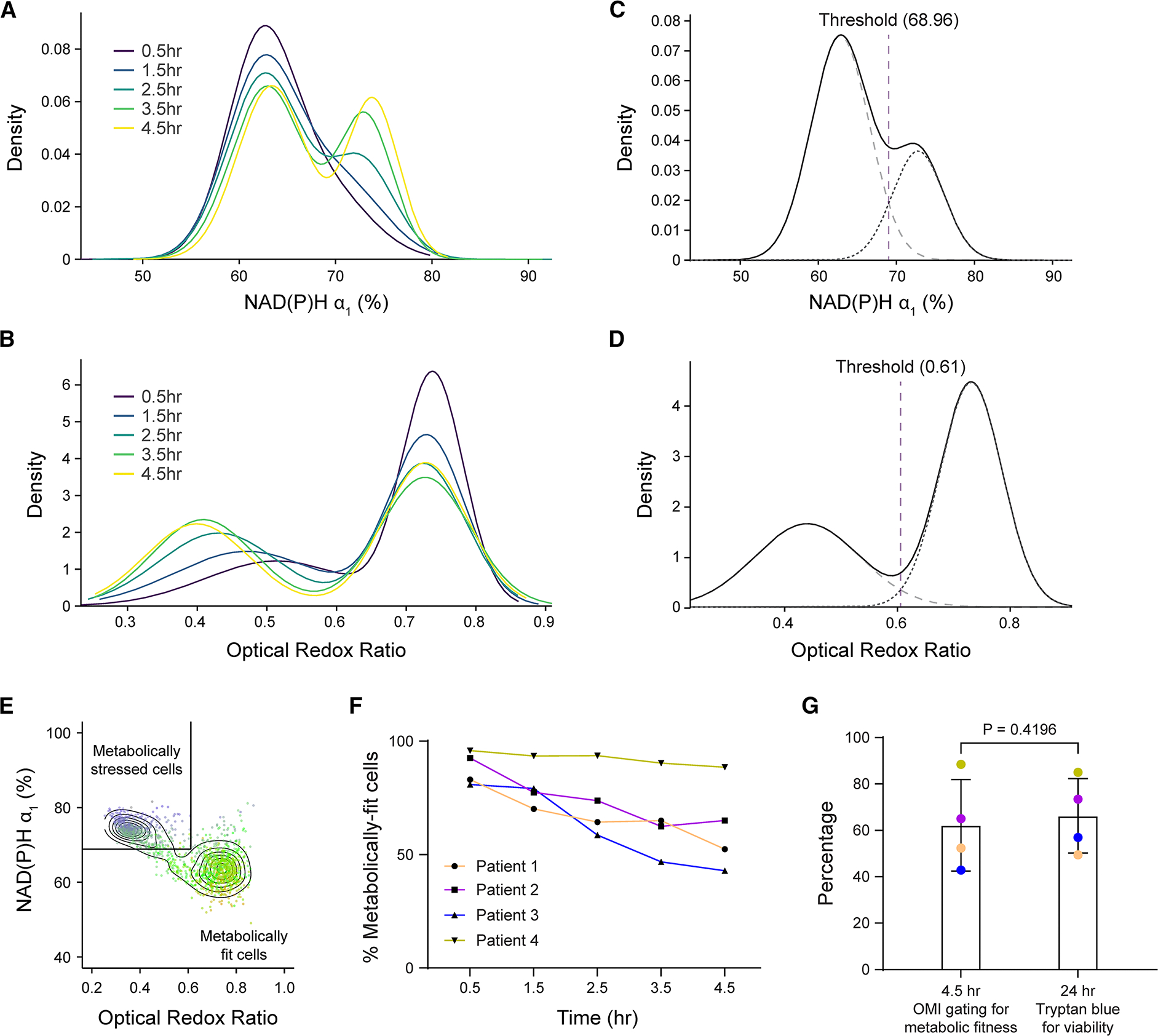
OMI provides an early measurement of recovery potential post-thaw in patient T cells (A and B) Density distribution of (A) NAD(P)H α_1_ and (B) ORR in patient T cells over a 4.5-h post-thaw time course. (C and D) Two-component Gaussian mixture models fit to pooled data across all time points from four patients for (C) NAD(P)H α_1_ and (D) ORR. Thresholds were determined as the intersection of the two fitted Gaussian distributions. *n* = 11,921 cells. (E) Gating strategy applied to NAD(P)H α_1_ and ORR to define metabolically stressed and fit cells. (F) Percentage of metabolically fit cells gated by OMI features (NAD(P)H α_1_ and ORR) over time post-thaw. (G) Percentage of OMI-gated metabolically fit cells at 4.5 h post-thaw and cell viability by Trypan blue staining at 24 h *n* = 4 patients. Two-sided paired *t* test.

We hypothesize that this population reflects an early metabolic stress response induced by cryopreservation and thawing. Density distribution of NAD(P)H α_1_ and ORR revealed the progressive transition from low-NAD(P)H α_1_ and high-ORR to high-NAD(P)H α_1_ and low-ORR in these patient T cells over 4.5 h upon thawing (Figures 6A and 6B). To identify these metabolic subpopulations, we applied a two-component Gaussian mixture model to the density distributions of NAD(P)H α_1_ and ORR pooled from four patients and defined the thresholds for each subpopulation as 68.96 for NAD(P)H α_1_ and 0.61 for ORR (Figures 6C and 6D). Cells within this gate (NAD(P)H α_1_ > 68.96% and ORR <0.61) were considered metabolically stressed, while those outside of this gate represented a metabolically fit population upon thawing (Figure 6E). Using this OMI-based gating, we observed a time-dependent decline in metabolic fitness within the first 4.5 h post-thaw from patient T cells (Figure 6F). Remarkably, metabolic fitness assessed by OMI at 4.5 h were consistent with viability measured by Trypan blue exclusion at 24 h in patient samples (Figure 6G). Hence, in this context, we defined “metabolic fitness” as the ability to recover and retain viability at 24 h post-thaw. This suggests that OMI could serve as an early, non-destructive indicator of T cell recovery potential post-thaw.

### OMI identified activation-associated metabolic changes in T cells from complete responders post-thaw

**Figure 7.**
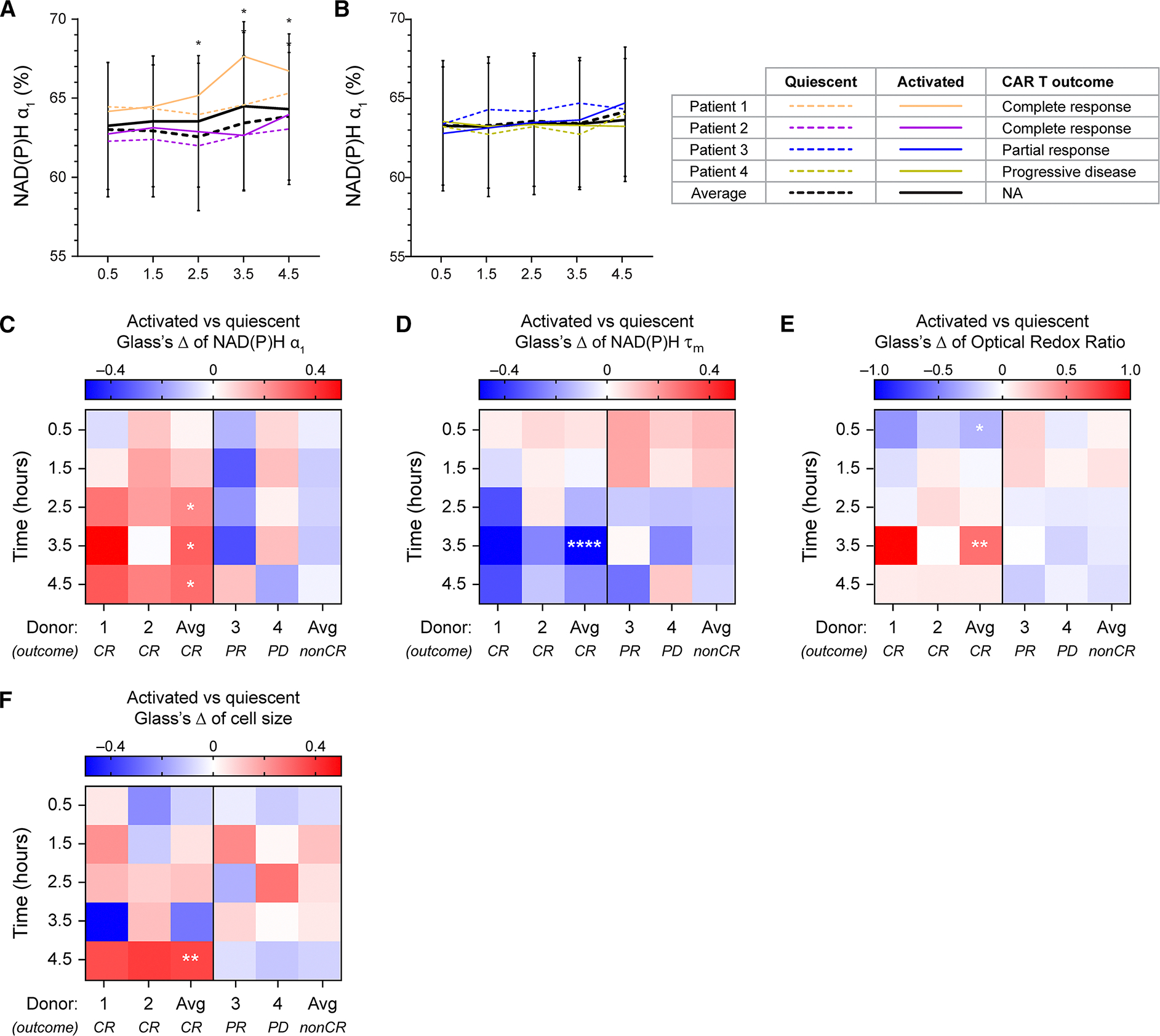
Metabolically fit T cells from patients achieving complete response to CAR T treatment demonstrated activation response post-thaw (A and B) NAD(P)H α_1_ of quiescent and activated T cells from starting materials of patients with (A) complete response (CR) or (B) partial response (PR) and progressive disease (PD) in response to later CD20/CD19 CAR T treatment. Lines represent donor average, color coded by donors, patterned by activation status (dashed line: quiescent, solid line: activated). *n* = 197–367 quiescent cells and 157–290 activated cells at each time point across two donors (patient 1 and patient 2) for (A); *n* = 257–576 quiescent cells and 314–571 activated cells at each time point across two donors (patient 3 and patient 4) for (B). (C–F) Glass’s Δs to quantify effect size of activation on (C) NAD(P)H α_1_, (D) NAD(P)H τ_m_, (E) ORR, and (F) cell size of metabolically fit T cells from four patients with respect to donor-matched quiescent group at each time point. *p < 0.05, **p < 0.01, ****p < 0.0001. Linear model with activation status as fixed effect and donor as blocking factor with Benjamin-Hochberg post-hoc test for multiple comparisons between activated and quiescent groups at different timepoints. For (A and B), error bars are standard deviation across two CR and two non-CR patients.

Prior studies have shown that the metabolism and function of patient T cells used as starting materials can influence clinical outcomes following CAR T cell therapy.^35^ Therefore, based on previously defined OMI features, we gated for metabolically fit T cells from starting materials of four patients and evaluated their response to stimulation with αCD2/αCD3/αCD28 during the early post-thaw period. Interestingly, metabolically fit T cells from two patients who later achieved complete response (CR) to CD20/CD19 CAR T cell treatment demonstrated activation signatures over the 4.5-h time course. Specifically, activated T cells from these patients showed significantly higher NAD(P)H α_1_ compared to donor-matched quiescent cells starting at 2.5 h post-thaw (Figures 7A and 7C, left), while no significant activation-associated changes in NAD(P)H α_1_ were observed in activated T cells from the two patients with later partial response (PR) or progressive disease (PD) (Figures 7B and 7C, right). Additionally, activated T cells from CR patients also exhibited decreased NAD(P)H τ_m_, increased ORR, and increased cell size compared to quiescent cells throughout the 4.5-h post-thaw time course (Figures 7D–7F, left). Previous studies have validated the association between these OMI features and activation response in T cells, suggesting that metabolically fit cells from CR patients in our pilot study cohort retained the ability to activate post-thaw.^30,31,36^ Meanwhile, T cells from PR and PD patients did not show these clear activation characteristics with no significant difference observed in activated versus quiescent groups, suggesting while these cells retained viability following the freeze-thaw process, they demonstrated impaired functions (Figures 7C–7F, right).

## DISCUSSION

Cryopreservation of biological samples is central in research and clinical translation of cell therapies, where the intricate manufacturing process requires transportation of cell products between the manufacturing facility and treatment site. While previous research on the impact of cryopreservation on the efficacy of CAR T cell therapy suggests that cryopreserved products offer comparable potency and treatment response, there have been some evidence of lower CAR T persistence in patients receiving frozen products.^37^ Additionally, several CAR T manufacturing protocols require freezing of the starting material before shipping to the manufacturing site; however, the impacts of cryopreservation on patient T cells prior to CAR T manufacturing have yet to be fully characterized. Previously, cryopreservation has been shown to impair metabolism and reduce immunomodulatory functions of mesenchymal stem cells due to heat-shock response, which potentially compromises their therapeutic benefits.^38^ Cryopreservation has also been shown to impair mitochondrial coupling efficiency and ATP production in leukocytes, while increasing dependence on glycolysis.^39^ However, these effects were characterized after overnight resting in leukocytes. Meanwhile, resting of cryopreserved natural killer cells overnight post-thaw improves their cytotoxic functions,^40^ suggesting that the period immediately post-thaw is a critical time window when cell function might be responding to cryopreservation/thawing impacts and sensitive to cryoinjury.

Since frozen T cell and CAR T cell products are often used immediately after thawing in both manufacturing and clinical settings, we used OMI, a label-free non-invasive imaging method, to characterize the impact of cryopreservation and thawing on T cell metabolism and activation response throughout the first 4.5 h post-thaw. Across three healthy donors, we showed a conserved metabolic response of T cells upon thawing with high NAD(P)H τ_m_ and low NAD(P)H α_1_ that was sustained up to 4.5 h. These shifts in OMI measurements indicate an increase in NAD(P)H binding activity, potentially due to cell metabolism ramping up after thawing. This finding aligns with prior studies showing broad metabolite upregulation in hepatocytes and increased NAD(P)H-dependent dehydrogenase activity in stem cells within a similar time window, supporting overall metabolic activation within the first 4–5 h post-thaw.^41,42^ Meanwhile, fresh quiescent T cells exhibited stable OMI measurements throughout the imaging time course. By focusing on this early post-thaw time frame, we have demonstrated the sensitivity of OMI for assessing rapid changes in single-cell metabolism post-thaw that may precede phenotypic or functional changes measured by conventional assays.

We hypothesized that T cell metabolism immediately upon thawing was not ready to support proper activation response. Compared to fresh T cells with detectable metabolic responses as early as 30 min post-activation, activation-associated OMI changes in frozen T cells were not observed until 2.5 h, supporting our hypothesis. Metabolic changes due to activation in frozen T cells then continued to persist through 4.5 h post-thaw, suggesting that by this time, frozen T cells have begun to recover from cryopreservation and are able to mount a measurable, though limited, functional response to stimulation. Our data are consistent with previous studies showing impaired immune response, specifically reduced cytokine production, in peripheral blood mononuclear cells post-cryopreservation using heat-inactivated fetal bovine serum (FBS) + 7.5% DMSO.^43^ This suggests that functional impairment due to cryoinjury may be robust across similar protocols despite the differences in DMSO concentrations and the use of heat-inactivated versus non-heat-inactivated FBS, though further research is needed to draw comparisons across other cryoprotective agents. Interestingly, while activation response in frozen T cells recovered at 48 h post-thaw, their expansion capacity throughout a 7-day expansion process remained lower than donor-matched fresh T cells, indicating that impairment in the early activation response could negatively impact T cell expansion.

Using frozen-CMV-specific T cells and HLA-matched CMV peptides, we demonstrated the impact of cryopreservation on both CD3-mediated and antigen-specific activations in T cells. We did observe faster and stronger metabolic changes, along with increased production of inflammatory cytokines in frozen-CMV-specific T cells activated with CMV peptide compared to CD3 antibody. Our findings indicate that OMI is sensitive to differences in T cell metabolic response toward two types of activating stimuli.

With single-cell resolution, OMI has revealed the metabolic heterogeneity within the patient samples and allowed gating for metabolically stressed versus metabolically fit cells that are more likely to recover and maintain viability post-thaw. Using OMI, we identified metabolic stress signals emerging in patient T cells upon thawing that were consistent with donor-matched viability measured using Trypan blue staining at 24 h. This suggests that these metabolic signals precede cell death and may serve as an early and sensitive indicator of post-thaw cell health, complementing standard viability assessments that often remain high immediately post-thaw but declines substantially by 24 h^44,45^ The metabolically stressed population displayed low ORR, which potentially indicate increased oxidative stress, a known cryoinjury phenomenon,^13,45^ and/or oxidative phosphorylation metabolism. However, increased NAD(P)H binding activity (i.e., low NAD(P)H α_1_), which is required for efficient NAD(P)H-dependent antioxidant functions^46,47^ and oxidative metabolism, was not observed in this subset. Therefore, these OMI signatures of the metabolically stressed population in patient T cells potentially suggest impaired antioxidant response or mitochondrial damage post-thaw, though further research is needed to reliably identify the mechanistic drivers of our observations. Additionally, OMI analysis of metabolically fit cells upon stimulation with activating antibodies reveals functional differences in T cells isolated from patients achieving CR compared to those obtained from PR/PD patients, although the sample size (*n* = 4 patients) is too low to claim the generalizability of these findings. Activation signatures, including high NAD(P)H α_1_, low NAD(P)H τ_m_, low ORR, and increased cell size, were observed in CR patient T cells while absent in those from PR/PD patients. This suggests that retention of viability might not be sufficient for functional response in T cells post-thaw and that metabolic features should be assessed to evaluate patient T cell quality for CAR T manufacturing. Additionally, these findings support the application of OMI as a label-free and single-cell analytical tool for continuous monitoring of the dynamic and heterogeneous response in patient T cells. As these cells were undergoing both metabolic stress due to freeze-thaw process and activation response to stimulating antibodies, OMI allowed functional characterization of each subpopulation that could otherwise be confounded in bulk population measurements. Overall, our data highlight the potential of OMI as a sensitive tool for characterizing patient T cell quality after cryopreservation and screening of patient T cell fitness for CAR T manufacturing. As the demand for CAR T therapy currently surpasses supply,^48^ there is a need to stratify patients who will likely achieve clinical response for CAR T therapy and those who might benefit from alternative treatment regimens. Since current Food and Drug Administration (FDA)-approved CAR T products are autologous, patient T cell fitness has been proposed as an important determinant for their clinical outcomes.^49^ The differences in post-thaw activation response between T cells from CR patients and PR/PD patients could serve as a predictive biomarker for patient stratification. However, these early findings are hypothesis-generating due to limited sample size. Therefore, larger and well-controlled clinical cohorts are required to validate these observations and support the clinical translation of OMI.

### Limitations of the study

There are limitations to this study. First, it is important to note that several cryopreservation and thaw protocols are used in the context of T cell manufacturing. Since this study focuses on evaluating cell functionality post-thaw, we did not exhaustively test and optimize for cryopreservation parameters, such as cryoprotective agent composition and cooling rate. T cells from healthy donors were cryopreserved in FBS + 10% DMSO to ensure broad relevance of our findings, as this represents a widely used cryopreservation protocol for immune cells. Meanwhile, T cells from patients with B cell malignancies were cryopreserved in cGMP-grade CryoStor CS5 to adhere to their clinical workflow. The use of different cryoprotectant formulations, cooling devices, and storage history in these two groups may influence post-thaw functionality and recovery of their respective T cells. Therefore, while we observed distinct metabolic responses of T cells from healthy donors and patients with B cell malignancies upon thawing, specifically the emergence of the metabolically stressed subpopulation in the patient cohorts, several biological and technical factors potentially contribute to these differences. Furthermore, since patient samples were collected and analyzed retrospectively following completion of the clinical trial, donor-matched fresh patient samples were not available for parallel analysis and comparison. Differences in baseline disease and treatment status among patients could influence their response to cryopreservation and activation post-thaw. Due to these limitations, further research with controlled experimental conditions across more healthy donors, a larger patient cohort, and additional CAR T models are warranted to validate our findings and evaluate how these factors impact T cell function and recovery potential post cryopreservation. While OMI provides single-cell label-free assessment of cell metabolism based on NAD(P)H and FAD autofluorescence, additionally metabolic and functional assays are also needed to obtain further mechanistic insights on the complex response of T cells post-thaw.

Our findings also focused on cryopreservation of peripheral T cells prior to CAR manufacturing, while cryopreserved final products for storage and distribution is a more common practice in the field. Hence, future studies will focus on characterizing frozen CAR T products to better understand its impact on their therapeutic efficacy and clinical outcomes. Finally, cryopreservation is also critical for scientific research and other medical applications besides cell therapy (such as *in vitro* fertilization or testing of potential pathogens).^50^ While we did observe significant antigen response from CMV-specific T cells upon thawing, future research will further investigate whether OMI is sensitive to the impacts of cryopreservation in these contexts.

## MATERIALS AND METHODS

### T cell isolation and culture

Healthy donors were recruited, and informed consent was collected from all donors under a protocol approved by the Institutional Review Board at the University of Wisconsin-Madison. CD3 T cells were isolated from peripheral blood of healthy donors using RosetteSep Human T cell enrichment cocktail (STEMCELL Technologies) following the manufacturer’s protocol. Briefly, peripheral blood was incubated with 50 μL/mL T cell enrichment cocktail, then diluted in PBS + 2% FBS and layered on Lymphoprep density gradient (STEMCELL Technologies) for separation using centrifugation. Isolated T cells were then divided into two groups for fresh culture or cryopreservation. For the fresh culture group, T cells were resuspended in ImmunoCult XF T cell expansion medium at 1 million cells/mL and cultured overnight at 37°C, 5% CO_2_. For cryopreservation, T cells were resuspended in FBS+10% DMSO at 1 million cells/mL. Cryopreservation vials containing 1 mL T cell solution were then placed in a freezing container (CoolCell LX Cell Freezing Container, Corning) to control for freezing rate (approximately −1°C/minute based on manufacturer’s characterization) and transferred to −80°C freezer to be frozen overnight (Figure S1).

### Isolation and cryopreservation of patient T cells

This study was approved by the Medical College of Wisconsin and Froedtert Hospital Institutional Review Board and FDA under IND 17518. Patient cells were collected by clinical leukapheresis (Table S1); CD4/CD8 selection was performed on a CliniMACS Prodigy instrument (Miltenyi Biotec, Bergisch Gladbach, Germany) by positive immunomagnetic separation; 100–150E6 CD4/CD8-positive cells were washed with PBS, and then resuspended in 4 mL CryoStor CS5 (Biolife Solutions, Bothell, WA). The resuspended cells were placed in a 4 mL cryovial and frozen at −80°C overnight in a Mr. Frosty device (Thermo Fisher Scientific, Waltham, MA) before transferring to liquid nitrogen for long-term cryopreservation.

### Thawing and activation of T cells

Cryopreservation vials were submerged in a water bath at 37°C for 30 s initially to quickly raise temperature. For healthy donor T cells, warm fresh ImmunoCult XF T cell expansion medium was added to the cryopreservation vial one drop at a time to dilute DMSO while providing fresh media to support cell recovery. When no ice crystals were visible, all T cells were then transferred into 10 mL of warm fresh ImmunoCult XF media, then centrifuged to wash out DMSO residue. T cells were then counted and plated at 200,000 cells in 200 μL of fresh media into a glass-bottom 96-well plate. Immediately upon thawing and plating, frozen T cells were either kept in plain ImmunoCult XF media (quiescent) or activated with 5 μL of StemCell αCD2/αCD3/αCD28 antibody (activated) (Figure S1). To maintain consistency with our bispecific CD20/CD19 CAR T manufacturing condition, T cells from patients with B cell malignancies were thawed following the same procedure using fresh warm TexMACs supplemented with 3% human serum and 200 U/mL interleukin-2 (IL-2) to wash out residual DMSO and activated with TransAct αCD3/αCD28 antibody.

For the antigen-specific experiment, HLA-A*0201-restricted anti CMV T cells were obtained from Celero (item #1049–5147JN21and item #1049–5085AP21). Upon arrival, T cells were kept in liquid nitrogen until used. T cell thawing was performed following a similar protocol as above. Briefly, RPMI + 2% FBS + 1% Pen/Strep was warmed up to 37°C, then added by drop into cryopreserved T cell vials. T cells were then washed once with fresh warm media and plated at a density of 1 million cells/mL. Immediately upon thawing, CMV-specific T cells were plated onto 35 mm glass bottom, poly-D-lysine-coated imaging dishes (MatTek) at a concentration of 200,000 cells/200 μL in ImmunoCult T cell expansion media. T cells were then stimulated with either 5 μL of StemCell αCD2/αCD3/αCD28 antibody or 1 μL iTAg Tetramer/APC–HLA-A*02:01 CMV pp65 peptide (NLVPMVATV) (MBL International) to generate antibody and CMV activated groups, respectively.

### T cell imaging with OMI

Thiry minutes after thawing and activation, T cells were imaged using OMI every hour up to 4.5 h in a stage top incubator (37°C, 5% CO_2_) (Figure S1) as previously described.^36^ OMI was performed on a multiphoton microscope (Ultima, Bruker) using an inverted laser-scanning microscope body (Ti-E, Nikon) equipped with an ultrafast tunable laser source (Insight DS+, Spectra Physics). Two-photon excitation of NAD(P)H and FAD were performed at 750 nm (2.5 mW) and 890 nm (4.5 mW), respectively, using a 40× water immersion 1.15 NA objective (Nikon) with 2.5× optical zoom. Other imaging parameters include 4.8 μs pixel dwell time, 60 s integration time, and image size of 256 × 256 pixels. NAD(P)H and FAD emission spectra were collected using GaAsP photomultiplier tubes (H7422, Hamamatsu) using a 440/80 nm and 550/100 nm bandpass filters, respectively. Fluorescence decays of NAD(P)H and FAD were acquired using time-correlated single-photon counting (TCSPC) electronics (SPC 150, Becker & Hickl GmbH) using Prairie View Software (Bruker). Allophycocyanin (APC)-conjugated CMV peptide was excited at 980 nm, and emission signal was collected using 690/50 nm filter. Fluorescence intensity and lifetime images of NAD(P)H and FAD, together with immunofluorescence images of APC-conjugated CMV peptide, were collected for each field of view (FOV), with 3–5 representative FOVs imaged per condition. The instrument response function was measured using second-harmonic generation signal from urea crystals excited at 890 nm.

### Image segmentation and OMI analysis

Fluorescence decay curves collected with TCSPC were fit to a double-exponential decay using the weighted-least square model in SPCImage software to determine pixel-wise lifetimes of free and protein-bound NAD(P)H and FAD. NAD(P)H and FAD lifetime images were binned to combine fluorescence counts from 25 neighbor pixels for fitting. Pixel-wise ORR was calculated as the normalized ratio between NAD(P)H fluorescence intensity and the sum of NAD(P)H and FAD fluorescence intensity. Individual cell and individual cytoplasm were segmented for CD3-mediated and antigen-specific experiments, respectively. CellPose cyto2 model was used for automatic segmentation of whole cells, with manual check by users to ensure accurate masking for each FOV. Thirteen OMI variables were collected and quantified, including ORR, fluorescence intensity, and mean fluorescence lifetime (τ_m_) of NAD(P)H and FAD, their free- and protein-bound lifetime components (τ_1_, τ_2_), and corresponding fractional contributions (α_1_, α_2_).

### Cytokine production analysis

Immediately post-OMI time course, T cell cultures were collected in a 1.5 mL Eppendorf tube, centrifuged at 300 g for 5 min, and spent media (supernatant) were collected for cytokine production analysis using Human TNF-alpha DuoSet ELISA kit (Bio-Techne) and Human IFN-gamma DuoSet ELISA kit (Bio-Techne) following manufacturer protocols. Cytokine production for each experimental group was quantified based on optical density read at 450 nm with wave-length correction at 570 nm.

### Statistical analysis

Statistical significance among experimental groups was performed in Prism (v.10.2.2) or R-Studio (v.3.5.3). Two-sided non-parametric Kruskal-Wallis test was used to eliminate bias and assumption of normality in the dataset. Dunn’s post hoc test was chosen to adjust for multiple comparisons. To determine the effect size of metabolic changes within the same treatment condition (e.g., frozen quiescent, frozen activated, fresh quiescent, or fresh activated group) over time, Glass’s Δ was calculated as

(Equation 1) $${\mathrm{Glass}}^{\prime }s\;\Delta_{ij}=\frac{\mu_{ij}\;-\;\mu_{i\;0.5hr}}{\sigma_{i\;0.5hr}}$$

where $\mu_{ij}$ is the mean value for the OMI parameter of interest for donor i at time point j, $\mu_{i0.5hr}$ is the donor-matched mean value of the corresponding OMI parameter at the 0.5 h time point, and $\sigma_{i0.5hr}$ is the standard deviation of $\mu_{i0.5hr}$ (Figures 1F, 1G, 2F, and 2G). To determine the effect size of activation on T cell metabolism at each time point,

(Equation 2) $${\mathrm{Glass}}^{\prime }s\;\Delta_{ij}=\frac{\mu_{{\mathrm{activated}}_{ij}}\;-\;\mu_{{\mathrm{quiescent}}_{ij}}}{\sigma_{{\mathrm{quiescent}}_{ij}}}$$

(Figures 3F, 3G, 7C–7F, S2C, and S4H). Glass’s Δ values were reported for individual donors, as well as the averaged values across three donors. To determine thresholds for subpopulations within the patient T cell samples, a two-component Gaussian mixture model was fit to the density distribution of OMI parameters, such that each component has at least 10% weight of the total population. Thresholds were determined as the intersection of the two component densities using Brent’s method for root finding routine.^51^

## Supplementary Material

1

SUPPLEMENTAL INFORMATION

Supplemental information can be found online at https://doi.org/10.1016/j.omta.2026.201704.

## Data Availability

All data are available upon request to the authors.
