# Supplementary material for: Autofluorescence imaging reveals the impact of cryopreservation on T cell metabolism and activation response
Source: Mol Ther Adv. Author manuscript; Available in PMC 2026 May 16. (PMC13148902; doi:10.1016/j.omta.2026.201704)
Supplement: 1 [file NIHMS2162492-supplement-1.pdf]

OMTA, Volume *34*

Supplemental information

Autofluorescence imaging reveals the impact of cryopreservation on T cell metabolism and activation response

Dan L. Pham, Meghana Kalluri, Cole Weaver, Angela Hsu, Amani Gillette, Wenxuan Zhao, Tyce Kearl, Peiman Hematti, Nirav N. Shah, and Melissa C. Skala

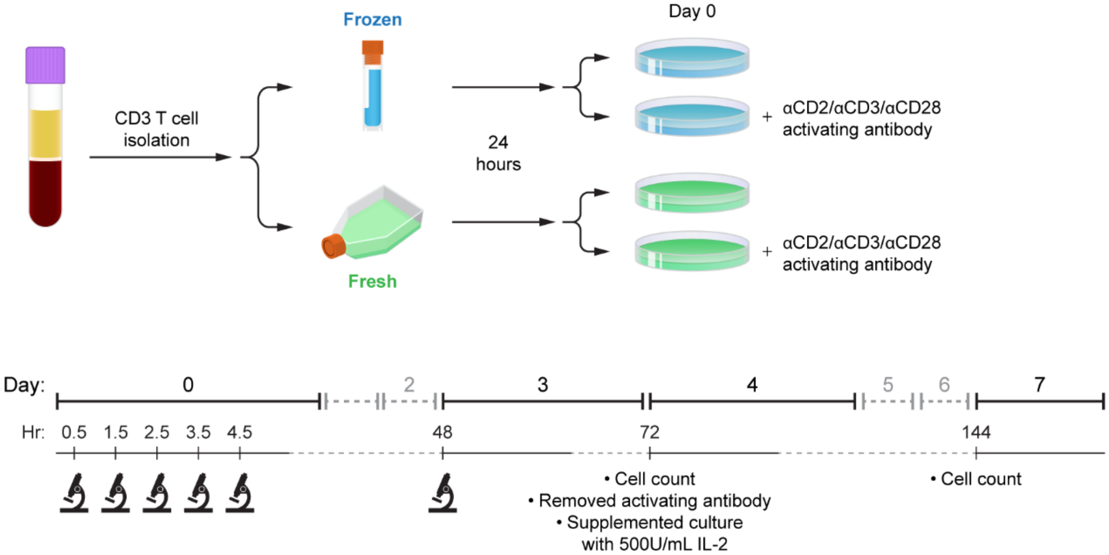

Figure S1. Experimental Setup. CD3 T cells were isolated from peripheral blood of healthy donors and divided into two groups for cryopreservation or fresh culture overnight. After 24 hours (day 0), cryopreserved T cells were thawed, and donor-matched fresh T cells were also harvested. Both frozen and fresh T cells were stimulated with αCD2/αCD3/αCD28 T cell activator and imaged with OMI every hour up to 4.5 hours. Fresh and frozen quiescent and activated cells were imaged again at 48 hours (day 2). 72 hours post activation (day 3), cells were counted and resuspended in fresh ImmunoCult XF T cell expansion media supplemented with 500U/mL IL-2. Cells were expanded up to day 7, when cell count was performed to determine fold expansion.

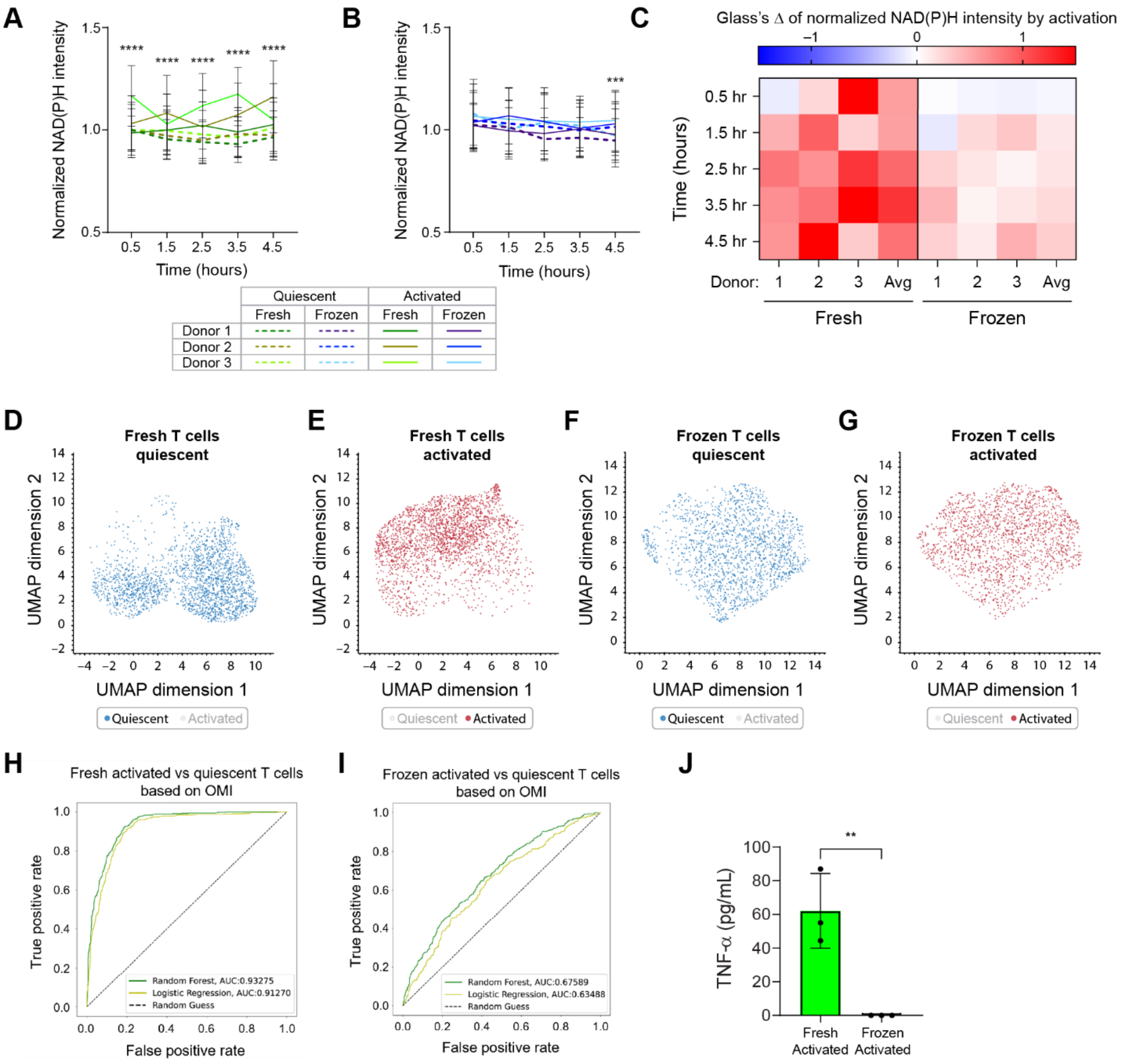

Figure S2. Frozen T cells demonstrated diminished activation response compared to donor-matched fresh T cells throughout 4.5-hour imaging time course. (A-B) Quantification of normalized NAD(P)H intensity of **(A)** fresh or **(B)** frozen quiescent and activated T cells from 3 independent donors. Lines represent donor averages, color coded by condition (fresh or frozen) and donor, line patterns indicate activation status (dashed line: quiescent, solid line: activated). NAD(P)H intensity at each time point was normalized to NAD(P)H intensity measurement of donor-matched quiescent fresh T cells at the 0.5-hour time point. n = 296-618 cells/condition/timepoint across 3 donors. ANOVA with three factors: donor (donor 1, 2, and 3), time (0.5-4.5 hours), and activation status (quiescent, activated). Tukey post hoc test was used to determine statistical significance for multiple comparisons between quiescent and activated groups at each time point. **(C)** Glass's Δs to quantify effect size of activation on normalized NAD(P)H intensity of fresh and frozen T cells over time, with respect to corresponding quiescent group at each time point. **(D-G)** UMAP of 11 OMI parameters (NAD(P)H and FAD τ_m, τ_1, τ_2, α_1, α_2, and

cell size) of **(D-E)** fresh and **(F-G)** frozen T cells from 3 donors based on activation status. n =3451-4595 cells. **(H-I)** Receiver operating characteristic (ROC) curves and areas under the curve (AUCs) of Random Forest (RF) and Logistic Regression (LR) algorithms to classify **(H)** fresh and **(I)** frozen T cells by activation status (activated versus quiescent) based on OMI parameters. Data were randomly split into 70% for training (*n* = 2416-3216 cells) and 30% for testing (*n* = 1035-1379 cells). **(J)** TNF-α secreted by donor-matched fresh and frozen activated T cells after 4.5 hours of stimulation, (*n* = 3 technical replicates). Bars are mean ± SD. ** $p < 0.01$, *** $p < 0.001$, **** $p < 0.0001$

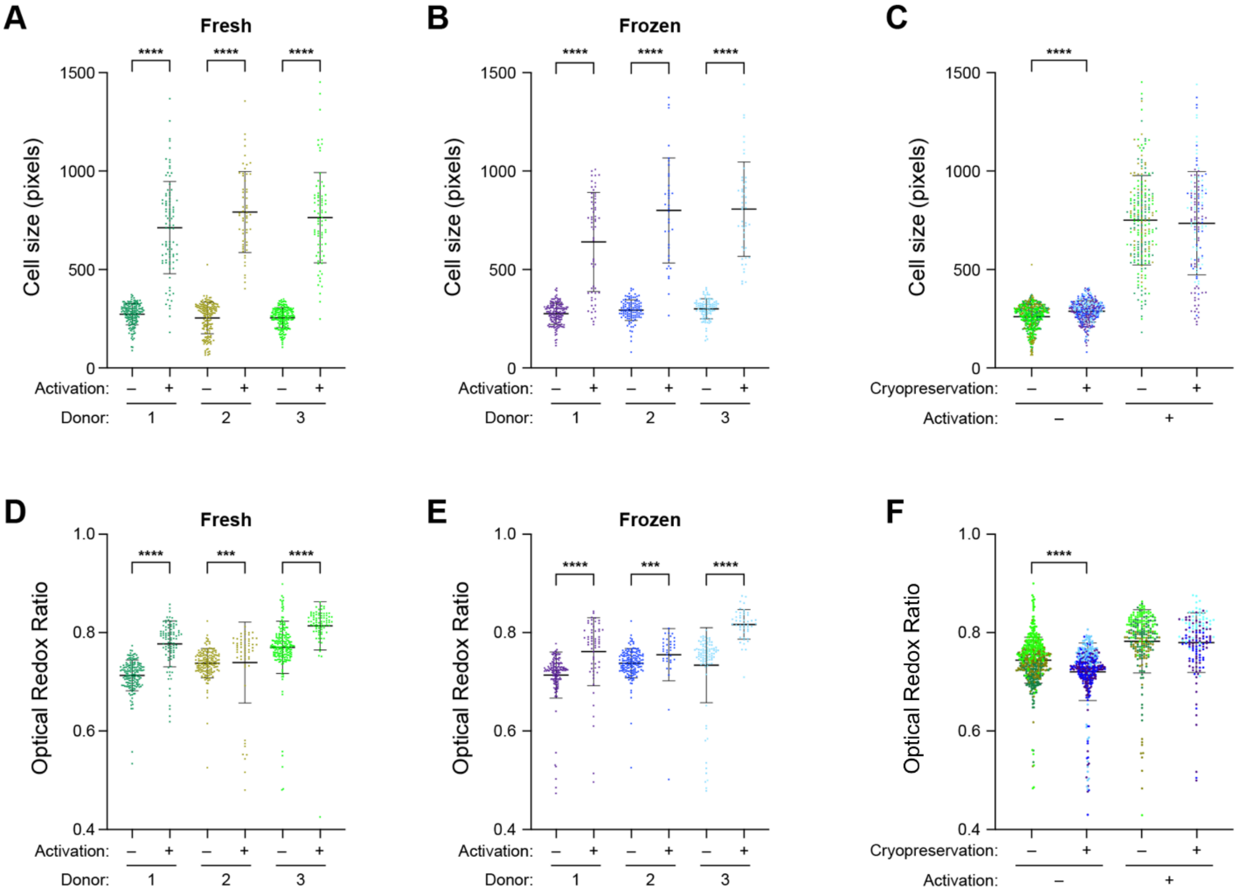

Figure S3. Activation response of fresh and quiescent T cells at 48 hours. **(A-B)** Quantification and **(C)** comparison of cell size of **(A)** fresh and **(B)** frozen quiescent and activated T cells at the 48-hour time point from 3 donors. **(D-E)** Quantification and **(F)** comparison of redox ratio of **(D)** fresh and **(E)** frozen quiescent and activated T cells at the 48-hour time point. For **(A-F)** n = 36-195 cells/condition/donor. Two-sided non-parametric Kruskal-Wallis test with Dunn's post hoc tests for multiple comparisons between **(A, B, D and E)** quiescent versus activated groups for each donor and **(C, F)** fresh versus frozen for all 3 donors. *** $p < 0.001$, **** $p < 0.0001$

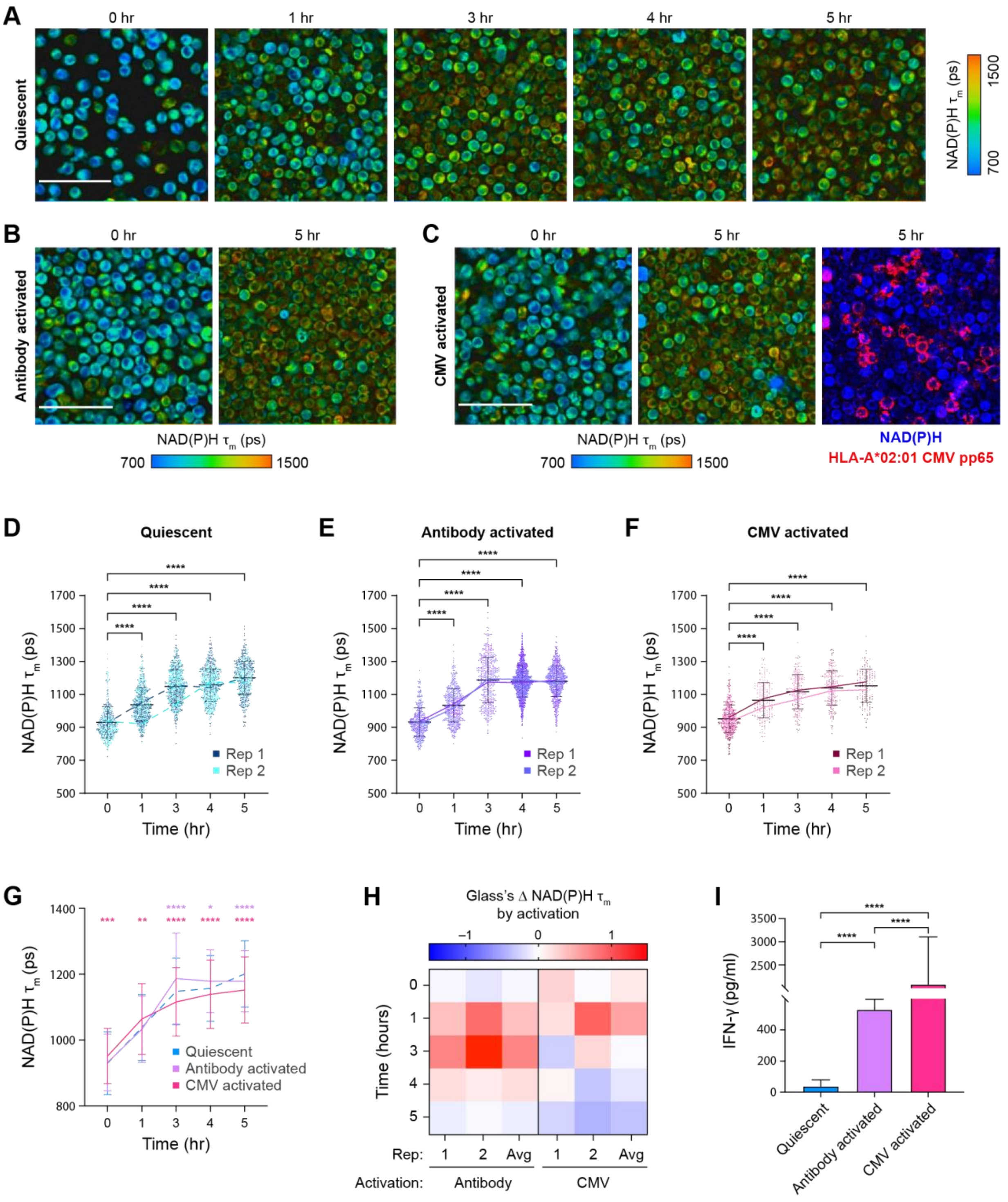

Figure S4. Antigen-specific activation response in frozen T cells. CMV-specific T cells were activated with two different activating stimuli: αCD2/αCD3/αCD28 antibody (antibody activation) or HLA-A*02:01 CMV pp65 peptide (CMV-peptide activation) upon thawing. **(A-C)** Representative NAD(P)H τ_m images of CMV-specific T cells in **(A)** quiescent, **(B)** antibody activated, or **(C)** CMV-peptide groups. APC-conjugated HLA-A*02:01 CMV pp65 peptide showed the presence of HLA-A*02:01 restricted CMV specific T cells (red). 0-hour images were

collected immediately post-thaw and before activation. **(D-F)** Quantification of NAD(P)H τ_m of **(D)** quiescent, **(E)** antibody activated, and **(F)** CMV-peptide activated CMV-specific T cells throughout 5-hour activation time course post-thaw. Only cells stained positive for APC-conjugated HLA-A*02:01 CMV pp65 peptide (red cells in panel **(C)**) were included in the analysis for the CMV-peptide activated group. n = 84-1172 cells/condition/time point across two biologically independent CMV-specific T cell batches (replicates) from one donor. Non-parametric Kruskal-Wallis test with Dunn's post hoc test for multiple comparisons against NAD(P)H τ_m measurements at the 0-hour time point. **(G)** Comparison of NAD(P)H τ_m between antibody activated and CMV-peptide activated T cells versus quiescent cells. Lines represent averages from two replicates of CMV-specific T cells from one donor. n = 84-1172 cells/condition/time point across 2 replicates. Two-way ANOVA with two factors: time (0, 1, 3, 4, 5 hours) and activation status (quiescent, antibody activated, and CMV-peptide activated). Dunnett's post hoc test for multiple comparisons of activation status (antibody activated versus quiescent, and CMV-peptide activated versus quiescent) at each time point. **(H)** Glass's Δ calculation for effect size of CD3-mediated (left) and antigen-specific (right) activation on NAD(P)H τ_m of cryopreserved CMV-specific T cells upon thawing. **(I)** Cytokine production by 200,000 CMV-specific T cells within 5 hours post-thaw with no stimulation (blue) or activated with αCD2/αCD3/αCD28 (purple) or HLA-matched CMV peptide (pink). n = 6 samples/condition across two batches of CMV-specific T cells. Kruskal Wallis test with Dunn's post hoc test for multiple comparisons. Scale bar is 50μm. Bars are mean ± standard deviation. * $p < 0.05$, ** $p < 0.01$, *** $p < 0.001$, **** $p < 0.0001$.

Table S1. Subject level demographic data and clinical outcomes[34]. DLBCL = diffuse large B-cell Lymphoma, MCL = mantle cell lymphoma. CR = complete response. PR = partial response. PD = progressive disease. Treatments: R=Rituximab, CHOP=cyclophosphamide, adriamycin, vincristine, prednisone, GDP=gemcitabine, dexamethasone, cisplatin, EPOCH=etoposide, prednisone, vincristine, cyclophosphamide, Adriamycin, ICE=ifosfamide, carboplatin, etoposide, BEAM=carmustine, etoposide, cytarabine, melphalan, DHAP=dexamethasone, cytarabine, cisplatin; BR=bendamustine-rituximab; MTX=methotrexate, Gem/Ox=gemcitabine, oxaliplatin.

Patient ID	Diagnosis	Prior Lines of Treatment	Day 28 Response
1	DLBCL<br>MYC+<br>t(8;14)	R-CHOP, Radiation, R-GDP, Rlenalidomide<br>Cell Therapy: axicabtagene ciloleucel 160 days prior to LV20.19 CAR	CR
2	DLBCL<br>MYC+<br>t(8;14)	R-EPOCH, R-GDP, Rlenalidomide	CR
3	DLBCL	R-CHOP, R-ICE<br>Transplant<br>BEAM auto-HCT	PR
4	MCL	R-CHOP/R-DHAP, BR, acalabrutinib, R-ICE, R-EPOCH, HD MTX, IT MTX, Gem/Ox<br>Transplant<br>BEAM auto-HCT	PD
